# Assessment of Ceftazidime-Avibactam 30/20-μg Disk, Etest versus Broth Microdilution Results When Tested against *Enterobacterales* Clinical Isolates

**DOI:** 10.1128/spectrum.01092-21

**Published:** 2022-01-12

**Authors:** Renru Han, Xuelin Yang, Yang Yang, Yan Guo, Dandan Yin, Li Ding, Shi Wu, Demei Zhu, Fupin Hu

**Affiliations:** a Institute of Antibiotics, Huashan Hospital, Fudan University, Shanghai, China; b The Second People’s Hospital of Tibet Autonomous Region of China, Tibet, China; c Key Laboratory of Clinical Pharmacology of Antibiotics, Ministry of Health, Shanghai, China; Emory University

**Keywords:** *Enterobacterales*, ceftazidime-avibactam, broth microdilution, Etest, disk diffusion

## Abstract

The objective of this research was to evaluate the correlation between inhibitory zones and MIC when testing ceftazidime-avibactam using disk diffusion, Etest, and broth microdilution method established by the Clinical and Laboratory Standards Institute (CLSI). Four-hundred and 58 isolates of *Enterobacterales* isolated from 54 medical centers from the China Antimicrobial Surveillance Network (CHINET) in 2016 to 2020 were collected. Antimicrobial susceptibility testing using broth microdilution, Etest, and disk diffusion were performed according to the CLSI. Of the 458 *Enterobacterales*, 17.2% (79/458) and 82.8%(379/458) were resistant or susceptible to ceftazidime-avibactam by broth microdilution, respectively. Compared with the broth microdilution method, the categorical agreement (CA) and essential agreement (EA) of the Etest were 99.6% (456/458) and 94.8% (434/458), respectively; the major error (ME) and very major error (VME) were both 0.2% (1/458). For disk diffusion, the CA and VME were 99.8% (457/458) and 0.2% (1/458), respectively. For Escherichia coli, the CA and EA of the Etest were 100% and 97.1% (135/139), respectively. The CA of the disk diffusion was 100%. For Klebsiella pneumoniae, the CA and EA of the Etest were 99.3% (288/290) and 93.4% (271/290), respectively, the ME and VME were both 0.3% (1/290). The CA and VME of disk diffusion were 99.7% (289/290) and 0.3% (1/290), respectively. For other *Enterobacterales*, the CA and EA of the Etest were 100% and 96.6% (28/29), respectively. The CA of the disk diffusion was 100%. Ceftazidime-avibactam disk diffusion (30/20-μg disks) and Etest demonstrated good performance for ceftazidime-avibactam susceptibility testing against *Enterobacterales* clinical isolates.

**IMPORTANCE** Multidrug-resistant Gram-negative bacteria, especially for extended-spectrum β-lactamases-producing and carbapenemase-producing *Enterobacterales*, are disseminating rapidly around the world. Treatment options for these infections are limited, which prompt the development of novel or combinational therapies to combat the infections caused by multidrug-resistant pathogens. The newly available β-lactam combination agent ceftazidime-avibactam has been demonstrated good *in vitro* and *in vivo* activity against ESBL, AmpC, KPC-2, or OXA-48-like-producing isolates and has shown promise in treating carbapenem-resistant *Enterobacterales* infections. Concerningly, there are few available automated systems for ceftazidime-avibactam susceptibility testing, and the broth microdilution method is hard to perform in most routine laboratories. Therefore, we urgently need an economical and practical method for the accurate detection of ceftazidime-avibactam activity against Gram-negative bacilli. Here, we evaluate the performance of the disk diffusion and Etest compared with the reference broth microdilution method against *Enterobacterales* clinical strains.

## INTRODUCTION

Multidrug-resistant Gram-negative bacteria, especially for extended-spectrum β-lactamases-producing and carbapenemase-producing *Enterobacterales*, are disseminating rapidly around the world, and the infections due to these pathogens cause high morbidity and mortality (1, 2). Treatment options for these infections are limited, which prompt the development of novel or combinational therapies to combat the infections caused by multidrug-resistant pathogens. Since 2015, the newly available β-lactam combination agent ceftazidime-avibactam has been approved for treating complicated urinary tract and intra-abdominal infections as well as hospital-acquired pneumonia and ventilator-associated pneumonia by the United States Food and Drug Administration (U.S. FDA).

Avibactam is a synthetic non-β-lactam β-lactamase inhibitor that inhibits the activities of Ambler class A, class C, and some class D enzymes, which broadens the spectrum of ceftazidime against β-lactamase-producing Gram-negative bacilli (1). As reported, ceftazidime-avibactam has been proved to have good *in vitro* activity against ESBL, AmpC, KPC-2, or OXA-48-like-producing isolates and has shown promise in treating carbapenem-resistant *Enterobacterales* infections (3–8). Several studies have demonstrated ceftazidime-avibactam might be an alternative option in treating multidrug-resistant *Enterobacterales* infections in combination with or without other antimicrobial agents (9–12).

To date, there are few available automated systems for ceftazidime-avibactam susceptibility testing, and the broth microdilution method is hard to perform in most routine laboratories. Thereby, we urgently need an economical and practical method for accurate detection of ceftazidime-avibactam activity against Gram-negative bacilli. Here, we evaluate the performance of the disk diffusion and Etest compared with the reference broth microdilution method against *Enterobacterales* clinical strains.

## RESULTS

The results of the broth microdilution method indicated that 82.8% (379/458) of isolates were susceptible to ceftazidime-avibactam, including 85 ESBL-positive, 91 ESBL-negative, 144 harboring *bla*_KPC-2_, 52 harboring *bla*_OXA-48-like_, four carbapenemase-negative, two harboring *bla*_IMP_, and one co-harboring bla_KPC_ and bla_NDM_. And 17.2% (79/458) clinical isolates were resistant to ceftazidime-avibactam including two ESBL-positive, three harboring *bla*_KPC-2_, three harboring *bla*_KPC-33_, six harboring *bla*_IMP_, 64 harboring *bla*_NDM_, and one co-harboring *bla*_KPC_ and *bla*_NDM_.

### The correlation between disk diffusion and broth microdilution.

**FIG 1:**
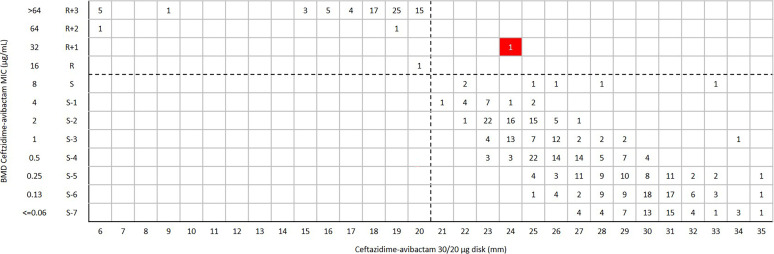
Scattergram comparing the results of ceftazidime-avibactam BMD MIC values (μg/mL) and disk diffusion zone diameters (mm) of a 30/20-μg disk against *Enterobacterales* isolates (*n* = 458). Dotted lines indicate ceftazidime-avibactam breakpoints (CLSI). The red background indicates that a very major error occurred for the disk diffusion method compared with the BMD.

**FIG 2:**
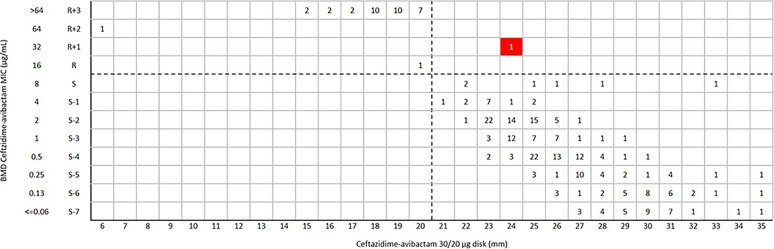
Scattergram comparing the results of ceftazidime-avibactam BMD MIC values (μg/mL) and disk diffusion zone diameters (mm) of a 30/20-μg disk against K. pneumoniae (*n* = 290). Dotted lines indicate ceftazidime-avibactam breakpoints (CLSI). The red background indicates that a very major error occurred for the disk diffusion method compared with the BMD.

**FIG 3:**
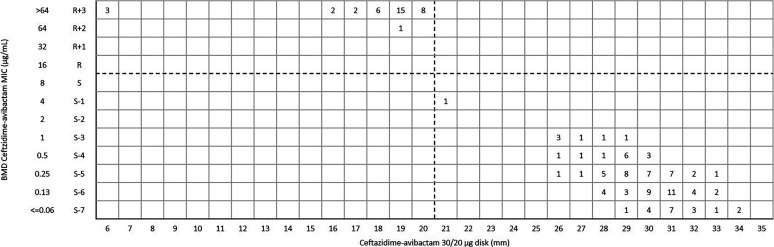
Scattergram comparing the results of ceftazidime-avibactam BMD MIC values (μg/mL) and disk diffusion zone diameters (mm) of a 30/20-μg disk against E. coli (*n* = 139). Dotted lines indicate ceftazidime-avibactam breakpoints (CLSI).

**FIG 4:**
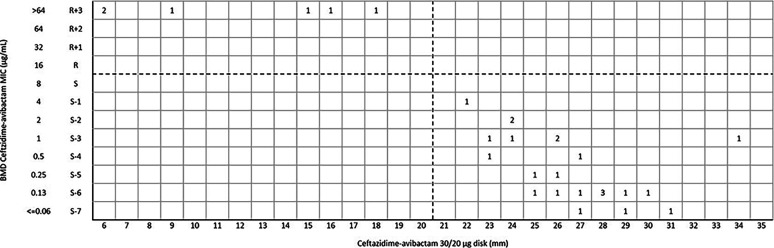
Scattergram comparing the results of ceftazidime-avibactam BMD MIC values (μg/mL) and disk diffusion zone diameters (mm) of a 30/20-μg disk against other *Enterobacterales* isolates (*n* = 29). Dotted lines indicate ceftazidime-avibactam breakpoints (CLSI).

For ceftazidime-avibactam disk diffusion, the categorical agreement (CA) and very major error (VME) were 99.8% (457/458) and 0.2% (1/458), respectively. One *bla*_KPC-2_-positive Klebsiella pneumoniae was susceptible to ceftazidime-avibactam (inhibition zone 24 mm) by disk diffusion, but the MIC was confirmed as resistant (MIC = 32 μg/mL) by broth microdilution method (BMD) (Fig. 1). Of 290 K. pneumoniae isolates, the CA and VME were 99.7% (289/290) and 0.3% (1/290) (Fig. 2), respectively. For Escherichia coli and other *Enterobacterales* (excluded E. coli and K. pneumoniae), the CA was 100% with no VME and major error (ME) (Fig. 3 and 4) (Table 1).

**TABLE 1:** Evaluation of agreement and errors among results of the Etest, disk diffusion, and BMD

Organism	MIC range	No. of isolates tested	E-test					Disk diffusion		
			No. (%) of CA	No. (%) of EA	No. (%) of VME	No. (%) of ME		No. (%) of CA	No. (%) of VME	No. (%) of ME
Escherichia coli	≥R + 1	37								
	R + S	0								
	≤S-1	102								
	Total	139	100	97.1(135/139)	0	0		100	0	0
Klebsiella pneumoniae	≥R + 1	35								
	R + S	7								
	≤S-1	248								
	Total	290	99.3(288/290)	93.4(271/290)	0.3(1/290)	0.3(1/290)		99.7(289/290)	0.3(1/290)	0
*Other Enterobacterales*	≥R + 1	6								
	R + S	0								
	≤S-1	23								
	Total	29	100	96.6(28/29)	0	0		100	0	0
*Total in Enterobacterales*	≥R + 1	78								
	R + S	7								
	≤S-1	373								
	Total	458	99.6(456/458)	94.8(434/458)	0.2(1/458)	0.2(1/458)		99.8(457/458)	0.2(1/458)	0

### The correlation between Etest and BMD.

**FIG 5:**
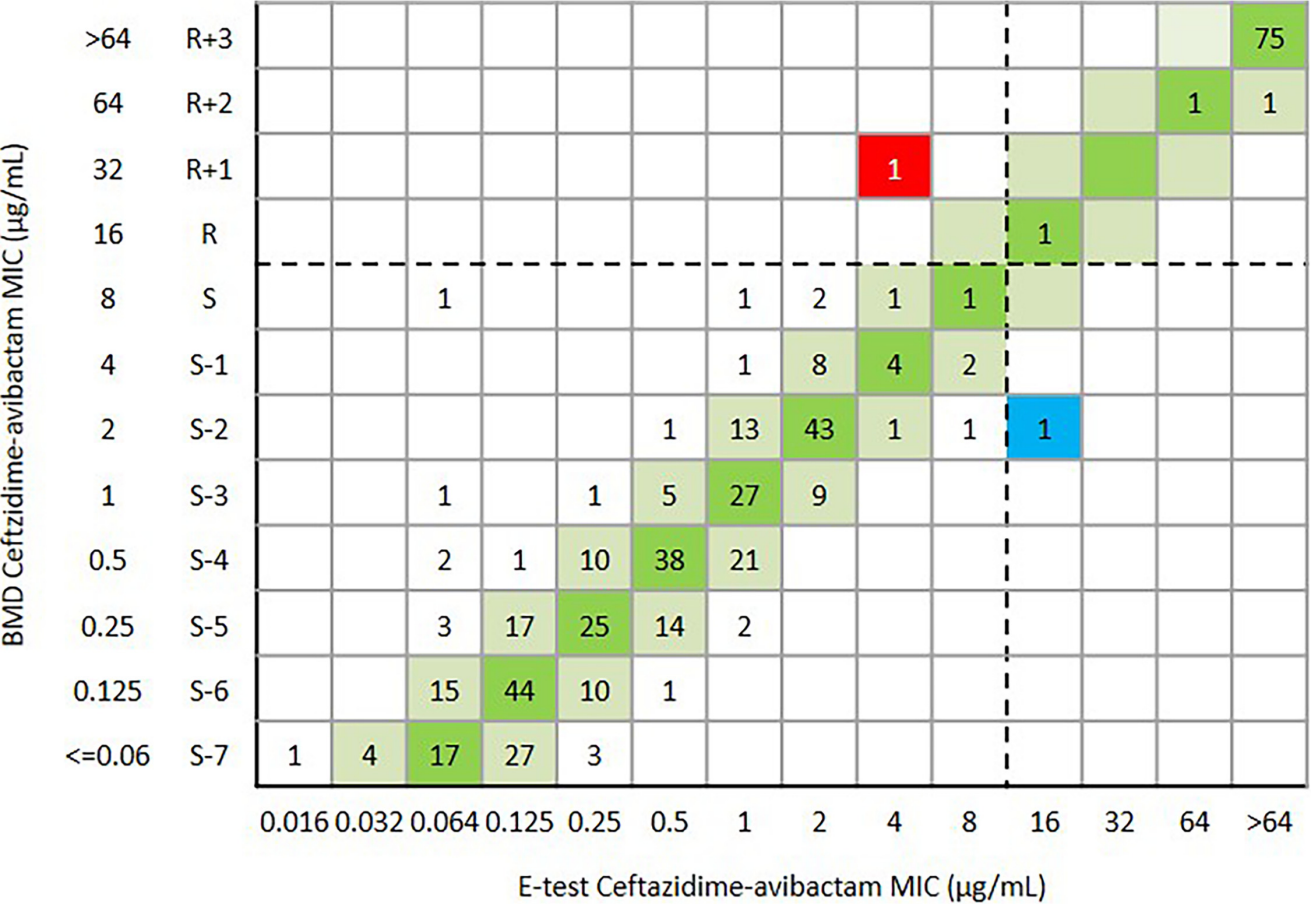
Scattergram comparing the results of ceftazidime-avibactam BMD MIC values (μg/mL) and Etest MIC values (μg/mL) against *Enterobacterales* isolates (*n* = 458). Dotted lines indicate ceftazidime-avibactam breakpoints (CLSI). The red and blue background indicates that a very major error and a major error occurred for the Etest compared with the BMD, respectively. The green and reseda background indicates that the EA of ceftazidime-avibactam BMD and Etest.

**FIG 6:**
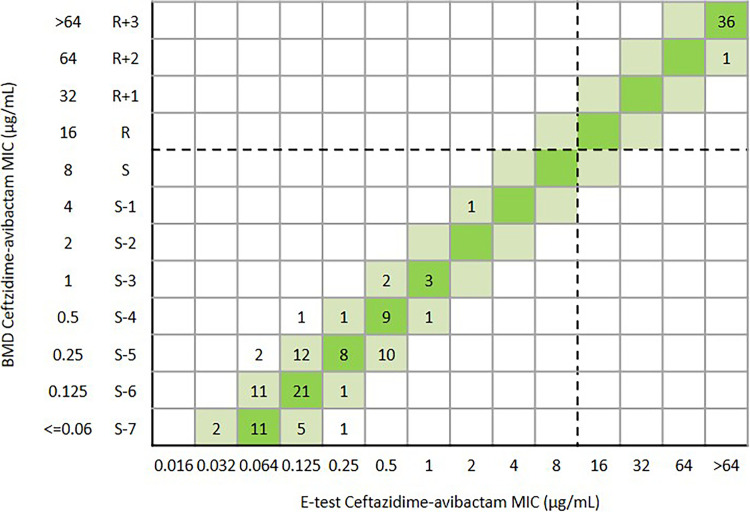
Scattergram comparing the results of ceftazidime-avibactam BMD MIC values (μg/mL) and Etest MIC values (μg/mL) against E.coli (*n* = 139). Dotted lines indicate ceftazidime-avibactam breakpoints (CLSI). The green and reseda background indicates that the EA of ceftazidime-avibactam BMD and Etest.

**FIG 7:**
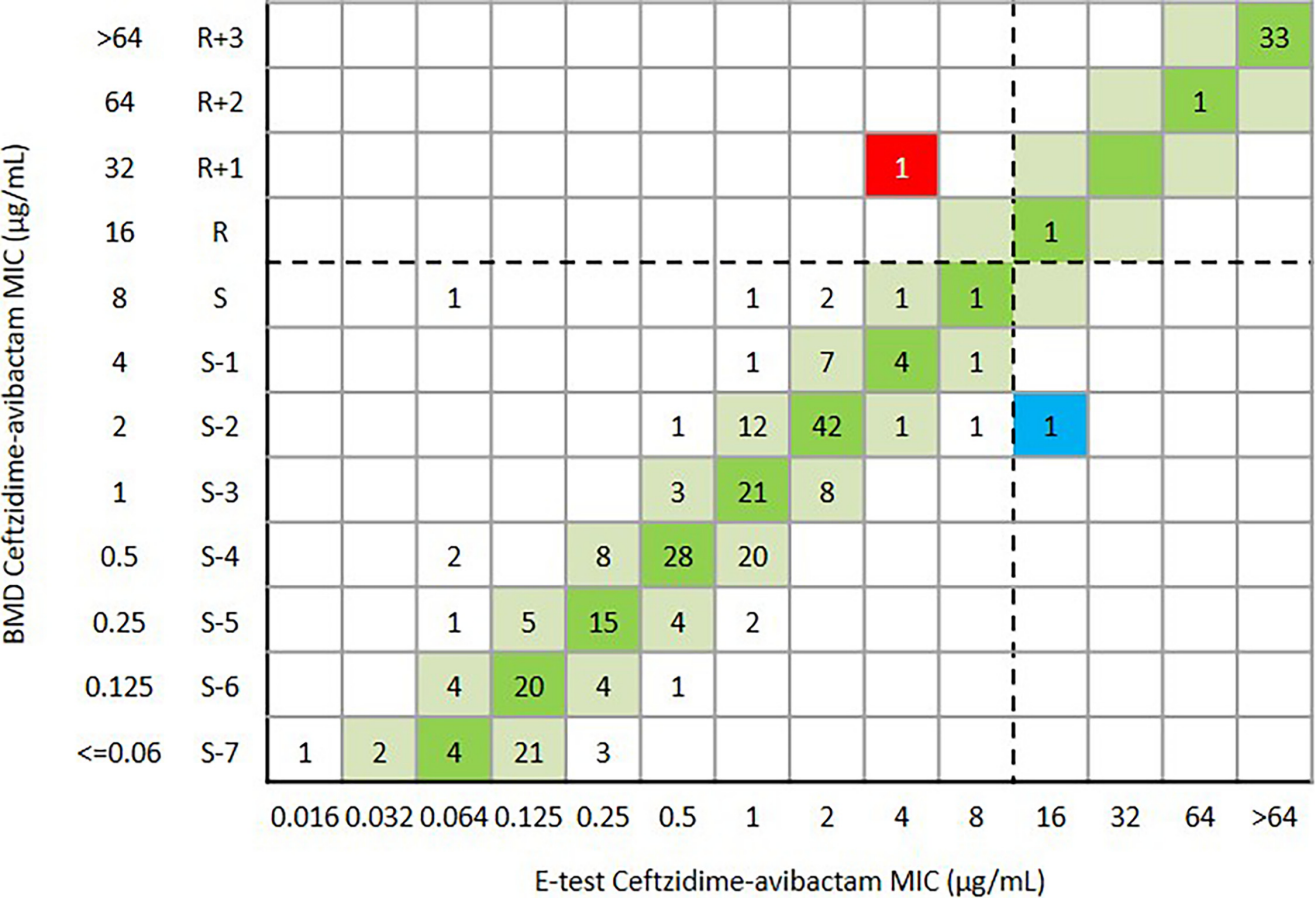
Scattergram comparing the results of ceftazidime-avibactam BMD MIC values (μg/mL) and Etest MIC values (μg/mL) against K. pneumoniae (*n* = 290). Dotted lines indicate ceftazidime-avibactam breakpoints (CLSI). The red and blue background indicates that a very major error and a major error occurred for the Etest compared with the BMD, respectively. The green and reseda background indicates that the EA of ceftazidime-avibactam BMD and Etest.

**FIG 8:**
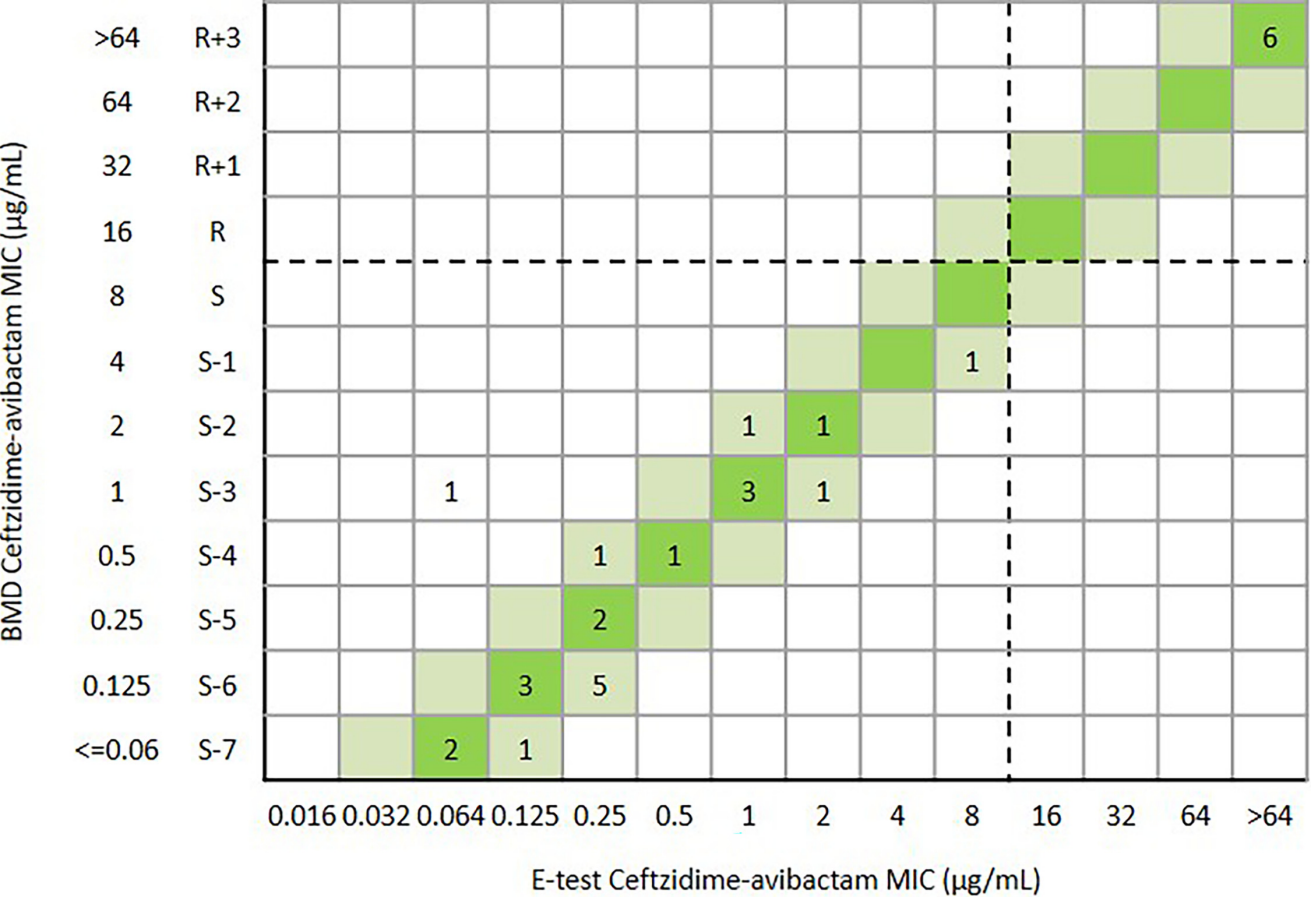
Scattergram comparing the results of ceftazidime-avibactam BMD MIC values (μg/mL) and Etest MIC values (μg/mL) against other *Enterobacterales* isolates (*n* = 29). Dotted lines indicate ceftazidime-avibactam breakpoints (CLSI). The green and reseda background indicates that the EA of ceftazidime-avibactam BMD and Etest.

Overall, the CA and essential agreement of the Etest were 99.6% (456/458) and 94.8% (434/458), respectively. The ME and VME were both 0.2% (1/458) (Table 1 and Fig. 5). One *bla*_KPC-2_-positive K. pneumoniae was susceptible to ceftazidime-avibactam (MIC = 4 μg/mL) by Etest, but the MIC was confirmed as resistant (MIC = 32 μg/mL) by BMD. Another *bla*_KPC-2_-positive K. pneumoniae was resistant to ceftazidime-avibactam (MIC = 16 μg/mL) by Etest but the MIC was confirmed as susceptible (MIC = 2 μg/mL) by BMD (Fig. 5). For 139 E. coli isolates, the CA and EA were 100% and 97.1% (135/139), respectively (Table 1 and Fig. 6). For 290 K. pneumoniae isolates, the CA and EA were 99.3% (288/290) and 93.4% (271/290), respectively. The ME and VME were both 0.3% (1/290) (Table 1 and Fig. 7). For 29 other *Enterobacterales*, the CA and EA were 100% and 96.6%(28/29), respectively (Table 1 and Fig. 8).

### The correlation between disk diffusion and BMD for isolates with ceftazidime-avibactam inhibitory zones of 20 to 22 mm.

In this study, 5.2% (24/458) isolates had the range of ceftazidime-avibactam inhibitory zones 20 to 22 mm, including nine of E. coli, 14 of K. pneumoniae, and one of Morganella morganii. For 16 isolates with inhibitory zones of 20 mm, in which 14 harboring *bla*_NDM_, one harboring *bla*_KPC_, and one harboring *bla*_IMP_, all of the isolates were confirmed as resistant with a MIC range of 16 to >64 μg/ml by BMD. For eight isolates with inhibitory zones of 21 to 22 mm, in which seven harboring *bla*_KPC_ and one carbapenemase-negative, all of the isolates were confirmed as susceptible with a MIC range of 2 to 8 μg/ml by BMD (Table 2).

**TABLE 2:** The correlation among disk diffusion, Etest, and BMD for isolates with ceftazidime-avibactam zones of 20 to 22 mm

Strain	Broth microdilution	E-test	Disk diffusion
E. coli	>64 μg/mL	>256 μg/mL	20 mm
E. coli	>64 μg/mL	>256 μg/mL	20 mm
E. coli	>64 μg/mL	>256 μg/mL	20 mm
E. coli	>64 μg/mL	>256 μg/mL	20 mm
E. coli	>64 μg/mL	>256 μg/mL	20 mm
E. coli	>64 μg/mL	>256 μg/mL	20 mm
E. coli	>64 μg/mL	>256 μg/mL	20 mm
E. coli	>64 μg/mL	>256 μg/mL	20 mm
K. pneumoniae	16 μg/mL	16 μg/mL	20 mm
K. pneumoniae	>64 μg/mL	>256 μg/mL	20 mm
K. pneumoniae	>64 μg/mL	>256 μg/mL	20 mm
K. pneumoniae	>64 μg/mL	>256 μg/mL	20 mm
K. pneumoniae	>64 μg/mL	>256 μg/mL	20 mm
K. pneumoniae	>64 μg/mL	>256 μg/mL	20 mm
K. pneumoniae	>64 μg/mL	>256 μg/mL	20 mm
K. pneumoniae	>64 μg/mL	>256 μg/mL	20 mm
K. pneumoniae	4 μg/mL	3 μg/mL	21 mm
E. coli	4 μg/mL	2 μg/mL	22 mm
K. pneumoniae	4 μg/mL	1.5 μg/mL	22 mm
K. pneumoniae	2 μg/mL	2 μg/mL	22 mm
K. pneumoniae	8 μg/mL	2 μg/mL	22 mm
K. pneumoniae	4 μg/mL	3 μg/mL	22 mm
K. pneumoniae	8 μg/mL	3 μg/mL	22 mm
Morganella morganii	4 μg/mL	6 μg/mL	22 mm

## DISCUSSION

Accurate and timely performance of antimicrobial susceptibility testing is crucial for the treatment of life-threatening infections such as carbapenemase-producing *Enterobacterales* (13–15). According to the Clinical and Laboratory Standards Institute (CLSI) guideline, confirmatory ceftazidime-avibactam MIC testing is indicated for isolates with zones of 20 to 22 mm to avoid reporting false-susceptible or false-resistant results. In this study, 5.2% (24/458) of isolates had ceftazidime-avibactam zones of 20 to 22 mm, including 14 of K. pneumoniae, nine of E. coli, and one of Morganella morganii (Table 2). The MIC were categorized as resistant with MIC ≥ 16 μg/ml for isolates with ceftazidime-avibactam zones of 20 mm. The MIC was also categorized as susceptible with MIC range 2 to 8 μg/ml for isolates with ceftazidime-avibactam inhibitory zones of 21 to 22 mm. In general, ceftazidime-avibactam 30/20-μg disk and Etest have performed very well when testing *Enterobacterales* isolates. Compared with BMD, the VME of ceftazidime-avibactam 30/20-μg disk was 0.2% and without ME, the VME and ME of ceftazidime-avibactam Etest were both 0.2% which were acceptable according to CLSI M23-A5. The CA of ceftazidime-avibactam 30/20-μg disk and ceftazidime-avibactam Etest were 99.8% and 99.6%, respectively. Ceftazidime-avibactam MIC and disk zone (30/20-μg disk) correlation were consistent with previous studies when testing *Enterobacterales* isolates (overall, VME and ME rates of 0.4% to 1.5% and 0.0% to 2.5%, respectively) (16, 17).

**FIG 9:**
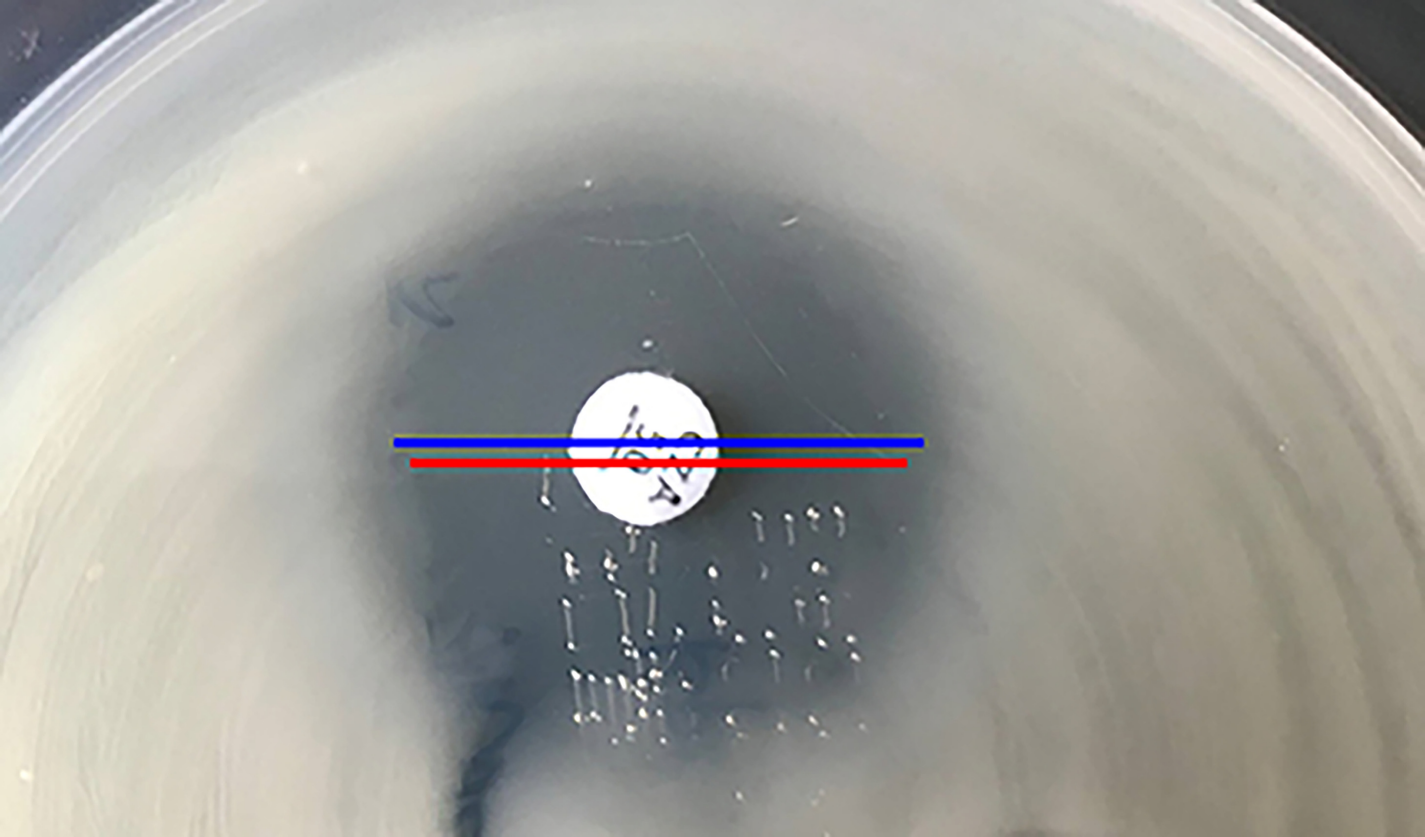
Ceftazidime-avibactam disk diffusion for K. pneumoniae. Disk diffusion inner (red line, 21 mm) or outer (blue line, 23 mm). The MIC of ceftazidime-avibactam was 4 mg/L confirmed by BMD. Ignore the thin veil of growth.

**FIG 10:**
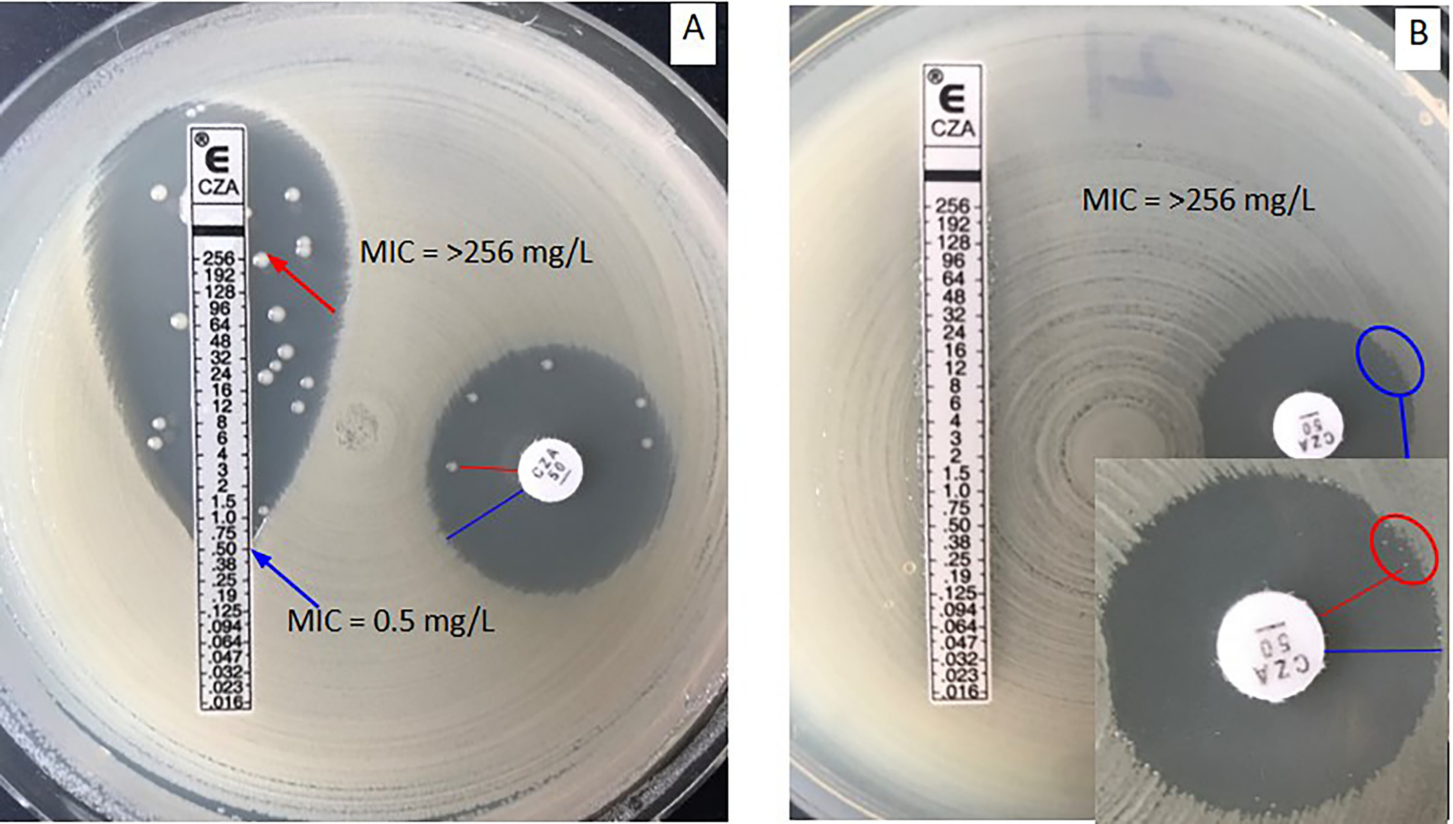
Ceftazidime-avibactam disk diffusion and Etest for K. pneumoniae (A) and Providencia rettgeri (B). (A) Disk diffusion inner (red line, 18 mm) or outer (blue line, 24 mm) and Etest inner MIC (red line, >256 mg/L) and outer MIC (blue line, 0.5 mg/L). (B) Disk diffusion inner (red line, 18 mm) or outer (blue line, 20 mm) and Etest MIC (>256 mg/L).

Several factors affect the susceptibility testing of ceftazidime-avibactam, including inoculum effect (18–20), and the measurement of the inhibitory zone, especially for isolates with ceftazidime-avibactam zones of 20 to 21 mm, because this zone range is borderline for the resistant and susceptible category. For disk diffusion, each ceftazidime-avibactam zone diameter should be measurable, and the zone diameter of complete inhibition including the diameter of the disk should be measured objectively with reflected light. It is important to note that the thin veil of growth in an otherwise obvious zone of growth inhibition should be ignored (Fig. 9). However, the growth of a single colony or multiple colonies within the inhibitory zone by disk diffusion or Etest should be considered and the inner margin should be measured to determine the ceftazidime-avibactam zone diameter (Fig. 10). Accurate measurement of ceftazidime-avibactam zone diameter is essential in routine work, as for precise infection treatment, and confirming MIC of ceftazidime-avibactam also needs to perform for these isolates with zone diameter 20 to 22mm according to CLSI (Fig. 10B).

Compared with CLSI guidelines (21), the European Committee on Antimicrobial Susceptibility Testing (EUCAST) (22) does not require the confirmatory MIC testing for *Enterobactarales* clinical isolates with ceftazidime-avibactam zones of 20 to 22 mm to avoid reporting false-susceptible or false-resistant results. In addition, the disk content for the disk diffusion test recommended by CLSI (30/20 μg) and EUCAST (10/4 μg) is diverse, and more studies need to evaluate the optimal ceftazidime-avibactam disk diffusion method, including disk content and the breakpoint because not all of the clinical microbiology laboratories have resources available for MIC testing (23).

## MATERIALS AND METHODS

### Clinical strains.

A total of 458 non-duplicate *Enterobacterales* strains were collected from China Antimicrobial Surveillance Network (CHINET, www.chinets.com/chinets) from January 2016 to July 2020, including K. pneumoniae (*n* = 290), E. coli (*n* = 139), Enterobacter cloacae (*n* = 10), Klebsiella aerogenes (*n* = 6), Citrobacter freundii (*n* = 6), M. morganii (*n* = 3), Klebsiella oxytoca (*n* = 2), Proteus vulgaris (*n* = 1), and Providencia rettgeri (*n* = 1). Of the 458 isolates, 280 were identified as carbapenem-resistant isolates (147 harboring *bla*_KPC-2_, three harboring *bla*_KPC-33_, two co-harboring bla_KPC_ and bla_NDM_, 64 harboring *bla*_NDM_, 52 harboring *bla*_OXA-48-like_, eight harboring *bla*_IMP_, and four carbapenemase-negative), 87 were extended-spectrum-β-lactamase positive isolates, and 91 were extended-spectrum-β-lactamase negative isolates. These clinical isolates were isolated from sputum (28.5%), urine (21.0%), blood (18.9%), bronchial-alveolar lavage fluid (8.6%), secreta (3.8%), bile (3.8%), pus (2.7%), abdominal fluid (2.4%), shunt fluid (2.4%), cerebrospinal fluid (1.4%), pleural fluid (1.4%), wound (1.0%), and other sources (4.1%). Most of the tested isolates were isolated from inpatients (93.7%) and a few from outpatients (6.3%). The most common departments were the intensive care unit (32.0%), neurosurgery department (6.6%), urology surgery department (5.5%), hepatobiliary surgery department (5.1%), infectious disease department (4.0%), general surgery department (4.0%), neurology department (3.7%), hematology department (3.7%), geriatric department (3.3%), respiratory medicine department (2.9%), outpatient and emergency department (6.3%), and other departments. All isolates were identified by MALDI-TOF/MS system (bioMérieux, France).

### Antimicrobial susceptibility testing.

All antimicrobial susceptibility tests for ceftazidime-avibactam were performed in parallel with disk diffusion (Oxoid, 30/20 μg), Etest (bioMérieux, France), and BMD in accordance with the Clinical and Laboratory Standards Institute (CLSI) reference method (21). To allow a fair comparison of the two methods, Etest MICs were rounded up to the next concentration when lying in between the standard values matching the 2-fold dilution scheme of the broth microdilution method. Mueller-Hinton agar (Oxoid, UK) plates were freshly prepared in each of the tests. The E. coli ATCC 25922 and K. pneumoniae ATCC 700603 were tested for quality control. The MICs and inhibitory zone diameter of ceftazidime-avibactam were interpreted using the breakpoints set by CLSI (MICs ≤8/4 μg/mL or zone diameter ≥21 mm were interpreted as susceptible, and MICs ≥16/4 μg/mL or zone diameter ≤20 mm were considered as resistant).

### Statistical analysis.

Essential agreement (EA) indicated that the Etest MIC agreed within ±1 log_2_ dilution with the BMD MIC. CA indicated that interpretive category results for the Etest method or disk diffusion method was the same as those for the reference BMD using CLSI breakpoints. VME indicated that the isolate was susceptible by the Etest or the disk diffusion but resistant according to the BMD. ME indicated that the isolate was resistant by Etest or the disk diffusion but susceptible according to the BMD. Very major discrepancy rates should be less than 1.5% and major discrepancies rates should be less than 3% when calculated based on all isolates according to CLSI M23-A5 (24).
